# Peripheral apoE isoform levels in cognitively normal *APOE* ε3/ε4 individuals are associated with regional gray matter volume and cerebral glucose metabolism

**DOI:** 10.1186/s13195-016-0231-9

**Published:** 2017-01-30

**Authors:** Henrietta M. Nielsen, Kewei Chen, Wendy Lee, Yinghua Chen, Robert J. Bauer, Eric Reiman, Richard Caselli, Guojun Bu

**Affiliations:** 1 Department of Neuroscience, Mayo Clinic College of Medicine, 4500 San Pablo Rd, Jacksonville, FL 32224 USA; 2 Department of Neurochemistry, Stockholm University, Svante Arrheniusväg 16B, SE-10691 Stockholm, Sweden; 3 Banner Alzheimer’s Institute, Phoenix, AZ 85012 USA; 4 Department of Mathematics and Statistics, Arizona State University, Tempe, AZ 85281 USA; 5 Arizona Alzheimer’s Consortium, Phoenix, AZ 85012 USA; 6 Department of Psychiatry, University of Arizona, Tucson, AZ 85721 USA; 7 Division of Neurogenomics, Translational Genomics Research Institute, Phoenix, AZ 85004 USA; 8 Department of Neurology, Mayo Clinic College of Medicine, Scottsdale, AZ 85259 USA

**Keywords:** Apolipoprotein E, Dementia, Plasma, Cognition, Gray matter volume

## Abstract

**Background:**

Carriers of the *APOE* ε4 allele are at increased risk of developing Alzheimer’s disease (AD), and have been shown to have reduced cerebral metabolic rate of glucose (CMRgl) in the same brain areas frequently affected in AD. These individuals also exhibit reduced plasma levels of apolipoprotein E (apoE) attributed to a specific decrease in the apoE4 isoform as determined by quantification of individual apoE isoforms in *APOE* ε4 heterozygotes. Whether low plasma apoE levels are associated with structural and functional brain measurements and cognitive performance remains to be investigated.

**Methods:**

Using quantitative mass spectrometry we quantified the plasma levels of total apoE and the individual apoE3 and apoE4 isoforms in 128 cognitively normal *APOE* ε3/ε4 individuals included in the Arizona *APOE* cohort. All included individuals had undergone extensive neuropsychological testing and 25 had in addition undergone FDG-PET and MRI to determine CMRgl and regional gray matter volume (GMV).

**Results:**

Our results demonstrated higher apoE4 levels in females versus males and an age-dependent increase in the apoE3 isoform levels in females only. Importantly, a higher relative ratio of apoE4 over apoE3 was associated with GMV loss in the right posterior cingulate and with reduced CMRgl bilaterally in the anterior cingulate and in the right hippocampal area. Additional exploratory analysis revealed several negative associations between total plasma apoE, individual apoE isoform levels, GMV and CMRgl predominantly in the frontal, occipital and temporal areas. Finally, our results indicated only weak associations between apoE plasma levels and cognitive performance which further appear to be affected by sex.

**Conclusions:**

Our study proposes a sex-dependent and age-dependent variation in plasma apoE isoform levels and concludes that peripheral apoE levels are associated with GMV, CMRgl and possibly cognitive performance in cognitively healthy individuals with a genetic predisposition to AD.

**Electronic supplementary material:**

The online version of this article (doi:10.1186/s13195-016-0231-9) contains supplementary material, which is available to authorized users.

## Background

More than two decades after the first report describing a significantly increased risk of developing late-onset Alzheimer’s disease (AD) in carriers of the *APOE* ε4 allele [1], this allele remains the strongest genetic risk factor for AD. Recent work further shows that the presence of the *APOE* ε4 allele also increases the risk of dementia with Lewy bodies (DLB) [2] whereas carriers of the *APOE* ε2 allele are protected against developing either of these two disorders [2, 3]. Although there is strong evidence that the presence of the *APOE* ε4 allele drives early and more abundant amyloid-beta (Aβ) pathology in AD, the exact biological mechanisms underlying the variation in risk of disease between the different *APOE* genotypes are not fully understood. Interestingly, cognitively healthy carriers of the *APOE* ε4 allele exhibit cerebral Aβ accumulation as early as in their third or fourth decade of life [4, 5]. Amyloid-β plaque pathology appears to be more prominent in female *APOE* ε4 carriers [4], who also are at a greater risk of AD than their male counterparts [6, 7]. Results from augmenting studies suggest an increase specifically in the pool of oligomeric Aβ in FAD-Tg mice expressing *APOE* ε4, in synaptosome-enriched preparations from *APOE* ε4 carriers and in *APOE* ε4 versus *APOE* ε3 carrier AD patients, of which the former AD patients exhibit higher levels of oligomeric Aβ surrounding amyloid plaques [8]. Consistent accumulating evidence further shows that *APOE* ε4 carriers devoid of cognitive symptoms exhibit low cerebral metabolic rate of glucose (CMRgl) in the posterior cingulate, precuneus, parietotemporal and frontal regions [9–13], and reduced regional gray matter volume (GMV) in the hippocampus [14] and amygdala [15], all brain regions known to be affected in AD. Interestingly, some of these changes can be found in young adults many years prior to the onset of cognitive dysfunction [16]. Even more compelling, these brain abnormalities appear to be present very early in life because recent results from a cross-sectional imaging study on infant *APOE* ε4 carriers (6–25 months of age) suggest that the white matter myelin water fraction and GMV are reduced in the precuneus, posterior/middle cingulate, lateral temporal and medial occipitotemporal regions compared with infant noncarriers [17]. These alterations may render *APOE* ε4 carriers more susceptible to AD-related pathology and predict conversion to AD in individuals with mild cognitive impairment (MCI) [18, 19]. Studies of *APOE* ε4-targeted replacement mice proposed a clear link between early-life stress and cognitive impairment in middle-aged mice, but whether these observations are of relevance to humans needs to be confirmed [20].

At the molecular level, various Aβ-dependent and independent mechanisms have been suggested to explain the increased risk of AD in *APOE* ε4 carriers [21]. For example, results from numerous studies on animal models show that the presence of the *APOE* ε4 allele promotes Aβ pathology [22]. In support, we and others have shown that the *APOE* gene product apolipoprotein E (apoE) negatively affects cellular Aβ uptake, for instance by glial cells [23–25]. In our work published recently we propose that cellular Aβ uptake is reduced in the presence of apoE due to competition between apoE and Aβ for binding to cell surface heparan sulfate proteoglycans (HSPGs) [26]. It has further been shown that animals expressing different human apoE isoforms exhibit APOE genotype-dependent variations in apoE levels, with the lowest apoE levels found in apoE4-expressing animals [27]. Other studies addressing apoE fluid concentrations in humans with different *APOE* genotypes, however, have reported conflicting results [28–31]. Technical issues were proposed to underlie the variation in reported apoE levels [28]. This is a valid concern because the three apoE isoforms only differ in one or two amino acid residues at positions 112 and 158, complicating the development of isoform-specific antibodies to be used in immuno-based quantification assays. To address this concern, we recently developed a specific multiplexed mass spectrometry-based assay for the absolute quantification of total apoE and individual apoE isoforms [32]. Employing this method we found that cerebrospinal fluid (CSF) and plasma levels of total apoE do not differ between AD patients and nondemented individuals [33]. However, plasma apoE levels were significantly reduced in *APOE* ε4 carriers with the lowest levels found in homozygous individuals, both controls and AD patients. As concluded from the results derived from *APOE* ε4 heterozygous individuals, the observed lower levels of plasma apoE in these individuals resulted from a specific reduction in the levels of the apoE4 isoform. In our previous study, total plasma apoE in heterozygous *APOE* ε4 was composed of roughly 30% apoE4 versus 70% non-apoE4 [33]. Because we found no difference in CSF or plasma apoE levels between AD patients and nondemented individuals we speculated that the peripheral apoE deficiency observed in *APOE* ε4 carriers may predispose these individuals to, rather than cause, neurodegenerative disease. An association between low levels of plasma apoE and risk of AD was suggested previously by findings from a prospective study of the Danish general population [34]. In the current study we aimed to quantify total plasma apoE as well as the individual apoE isoform levels and their effect on cognition, CMRgl and GMV in cognitively healthy *APOE* ε3/ε4 carriers. To our knowledge, this is the first report investigating whether the levels of individual apoE isoforms in plasma from *APOE* ε3/ε4 carriers are associated with GMV and CMRgl, as determined using structural and functional brain imaging, and cognitive performance.

## Methods

### Study cohort

The included individuals are a sample from the Arizona *APOE* Cohort for which recruitment and enrollment strategies have been described previously [35–37]. Briefly, the Arizona *APOE* Cohort consists of cognitively normal individuals residing in Maricopa County, AZ, USA. Since 1994, residents aged 21 years and older have been recruited through local media advertisements for inclusion in a study of cognitive aging. The Arizona APOE Cohort study subjects underwent *APOE* genotyping and agreed to have the test results withheld from them as part of the study protocol. To be considered for inclusion, participants had to score at least 27 points on the Mini-Mental State Examination and exhibit no evidence of depression (10 points or less on the Hamilton Depression Rating Scale). None of the included individuals met the published criteria for MCI [38] or AD [39] at study entry. Study subjects were followed and seen in the clinic every 1–2 years. Blood for plasma analysis was collected in tubes containing EDTA. Samples were centrifuged at 2000 × *g* at 4 °C for 10 minutes. Centrifuged samples were aliquoted and immediately frozen at –80 °C in polypropylene vials pending biochemical analysis. In the current study, 128 individuals with an *APOE* ε3/ε4 genotype from whom blood samples were drawn at the same visit or within 1 year of their neuropsychological assessment (see later) were included. Within this group, 25 individuals had undergone imaging (FDG-PET and MRI) plasma sampling and cognitive testing within the same year. All individuals gave informed written consent to participate in the study and the study protocol was approved by the institutional review boards of Banner Good Samaritan Medical Center (now Banner-University Medical Center, Phoenix, AZ, USA) and the Mayo Clinic.

### ApoE quantification

Plasma total apoE, apoE3 and apoE4 isoform concentrations were quantified using a previously described mass spectrometry-based multiplex selected reaction monitoring (SRM) assay [32] and the services provided by the Mayo Clinic Proteomics Core. Briefly, two tryptic peptides derived from the two major APOE single nucleotide polymorphisms (SNP112 and SNP158) were employed for the quantification of apoE3 and apoE4 isoforms (LAVYQAGAR and LGADMEDVR). As a control, total apoE concentrations were quantified in parallel using a tryptic peptide with a sequence present in all three known apoE isoforms (LGPLVEQGR). Plasma samples were diluted 1:100, digested as described previously [32] and analyzed using liquid chromatography–tandem mass spectrometry (LC-MS/MS) with a Waters NanoAcquity coupled to a Thermo Vantage mass spectrometer.

### Brain imaging

T1-weighted, three-dimensional, pulse sequence MRI and FDG-PET data were obtained for each subject (*n* = 25) as described previously [40]. All MRI scans were performed on the same 1.5-T Signa system (General Electric, Milwaukee, WI, USA) for the T1-weighted, three-dimensional pulse sequence (radiofrequency-spoiled gradient recall acquisition in the steady state (SPGR), repetition time = 33 ms, echo time = 5 ms, α = 30°, number of excitations = 1, field of view = 24 cm, imaging matrix = 256 × 192, slice thickness = 1.5 mm, scan time = 13:36 min). The reconstructed images included 128 contiguous horizontal MRI slices with slice thicknesses of 1.5 mm and in-plane voxel dimensions of 0.94 mm × 1.25 mm.

For FDG-PET, our study personnel consistently instructed participants to lie quietly with their eyes closed in the darkened scanner room. All PET scans were performed on the same HR+ scanner (Siemens, Knoxville, TN, USA) in the three-dimensional mode. The procedure included a transmission scan, an intravenous injection of 5–8 mCi of FDG and a 60-minute dynamic sequence of emission scans. The reconstructed images consist of 63 horizontal slices with a center-to-center slice separation of 2.46 mm, an axial field of view of 15.5 cm, an in-plane resolution of 4.2–5.1 mm full width at half-maximum and an axial resolution of 4.6–6.0 mm full width at half-maximum. PET images (counts relative to the whole-brain uptake) acquired during the last 30 minutes were used for the voxel-based analyses.

The automated brain mapping algorithmic program voxel-based morphometry (VBM), implemented in Statistical Parametric Mapping (SPM8; Wellcome Trust Centre for Neuroimaging, London, UK), was used to process the volumetric T1-MRI data, characterizing the gray matter spatial distributions and spatially deforming the individual gray matter maps to the predefined coordinate space, as defined by the Montreal Neurological Institute (MNI) brain template [41]. SPM8 was also used to spatially deform the individual FDG-PET data to the same MNI brain template.

General linear model-based, voxel-wise analysis examined the association of the apoE4/apoE3 ratio with volumetric MRI-based regional GMV or with FDG-PET-measured CMRgl respectively. For exploratory analysis, uncorrected *p* = 0.005 was chosen for all imaging-related analyses. This value has been shown to provide the optimal balance between type-I and type-II errors [42]. To adjust significance levels for the number of resolution elements in a-priori regions known to be affected by AD, previously characterized independently for volumetric MRI and FDG-PET from the Alzheimer’s disease Neuroimaging Initiative (ADNI) project, the association in each of the regions of interest was corrected for multiple comparisons with family-wise error (FWE) correction. A threshold of *p* < 0.05 was used via the small volume correction (SVC) procedure in SPM

### Plasma lipid profiling

The majority of the imaged subjects (*n* = 24) had undergone plasma lipid profiling including plasma total cholesterol, triglycerides and HDL and LDL cholesterol as part of the clinical work-up. Plasma lipids were assessed as described previously [43].

### Neuropsychological testing

The neuropsychological test battery administered to the study participants is described in detail elsewhere [37]. In addition to tests assessing global cognition (the Mini-Mental State Examination, the Instrumental Activities of Daily Living and the Structured Psychiatric Interview from the revised DSM III third edition), more specialized tests assessing four cognitive domains including memory, language, visuospatial and executive functions were administered. Tests and summary of results for the study subjects included in the present study are available in Additional file 1: Table S1.

### Statistical analysis

Normal distribution of the data was assessed using the Shapiro–Wilk *W* Test. Cognitive test data and total apoE, apoE3 and apoE4 levels were not normally distributed, hence nonparametric tests were used throughout the statistical analyses. Regression analysis was used to assess statistical associations between imaging data and apoE levels. If not stated differently, data are presented as median with range (minimum–maximum) whereas descriptive data, lipid profiles and cognition test results are shown as mean ± standard deviation.

## Results

### Study subject characteristics

A total of 128 individuals, 71% females, were included in this study. A subset of these individuals (*n* = 25) had undergone brain imaging studies. The demographics of the whole study cohort and imaged group are summarized in Table 1. In the whole cohort, the average age was slightly higher in the male group (*p* = 0.03), who also exhibited a trend toward higher body mass index compared with the females (*p* = 0.09). The female group differed from the male group having 6.7% more non-Caucasian study participants. The group of females versus males did not differ with regard to incidence of diabetes, family history of dementia and average years of education. No difference was observed between parameters of the whole cohort and the imaged subgroup.

**Table 1:** Study subject characteristics

	*N*	Age at investigation (years)	Race (% White)	Body mass index	Diabetes (% yes)	Dementia family history (% yes)	Education (years)
Total	128	63.1 ± 10.6	89.8	26.1 ± 4.4	3.1	81.3	15.9 ± 2.3
Females	91	61.8 ± 10.4	87.9	25.8 ± 4.8	2.2	81.3	15.9 ± 2.3
Males	37	66.3 ± 10.5	94.6	27.0 ± 3.3	5.4	81.1	16.0 ± 2.3
Imaged subjects	25	65.7 ± 5.0	88.0	26.7 ± 3.8	8	100	16 ± 2.6
Females	17	65.2 ± 5.3	82.4	25.7 ± 4	0	100	15.8 ± 2.8
Males	8	64.4 ± 5.6	100	28.7 ± 2.5	25	100	16.6 ± 2.3

Data presented as mean ± standard deviation or percentage

### Plasma levels of total apoE and individual apoE3 and apoE4 isoforms

**Fig. 1:**
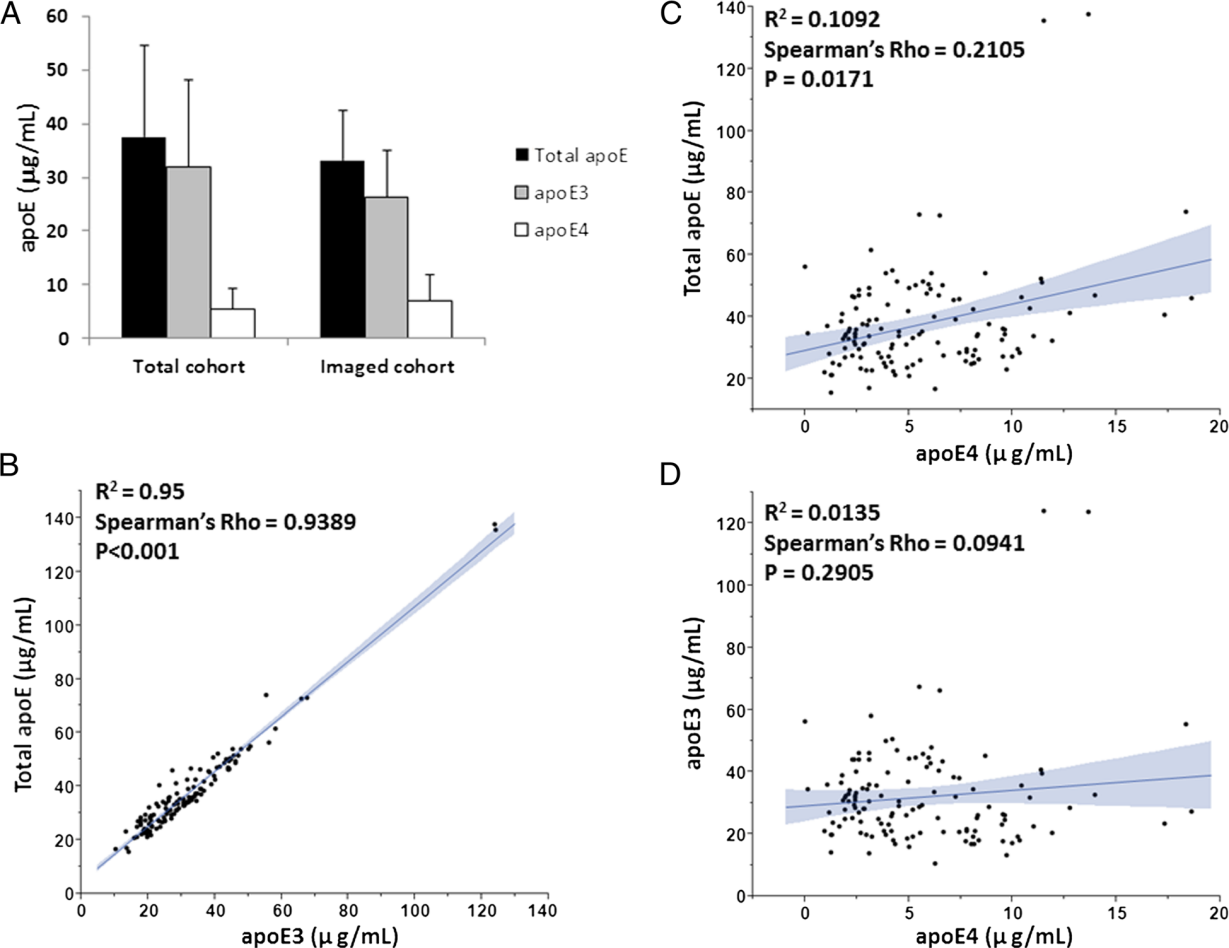
Plasma apoE levels. Plasma levels of total apoE, apoE3 and apoE4 isoforms in the total *APOE* ε3/ε4 cohort studied (*n* = 128) and in the imaged cohort (*n* = 25) (**a**). Correlation between plasma total apoE levels and the individual apoE3 (**b**) and apoE4 (**c**) isoform levels, and between plasma apoE3 and apoE4 isoform levels (**d**). *apoE* apolipoprotein E

Total plasma apoE concentrations were similar in the whole cohort and in the imaged subgroup (Fig. 1a), and consisted of 78.9% versus 85% apoE3 isoform and 21% versus 15% apoE4 isoform (Fig. 1a). Total apoE was strongly and positively correlated to apoE3 levels (Fig. 1b) in both groups (separate results for imaged group not shown), whereas apoE4 levels only weakly associated with total apoE levels (Fig. 1c) (*n* = 128). Importantly, we found no significant correlation between the plasma concentrations of the apoE3 and apoE4 isoforms (Fig. 1d). We further found no association between plasma levels of total apoE and individual apoE isoforms, age at investigation, body max index (BMI), family history of dementia, incidence of diabetes or total years of education (data not shown). Because apoE is an important lipid carrier both in the periphery and in the CNS, we examined the lipid profiles obtained from a subset of individuals (*n* = 24) to assess potential relationships with apoE. Lipid profiles are presented in Table 2. Total apoE levels were positively associated with total plasma cholesterol levels (Spearman’s ρ = 0.6552, *p* = 0.0005) and this association was driven by the apoE3 isoform levels (Spearman’s ρ = 0.6744, *p* = 0.0003). Total plasma apoE levels were also positively related to plasma LDL cholesterol levels (Spearman’s ρ = 0.4319, *p* = 0.0351), which is similar to the link between apoE and total cholesterol driven by apoE3 levels (Spearman’s ρ = 0.4806, *p* = 0.0174). We found no statistically significant associations between apoE4 isoform levels and any of the plasma lipids.

**Table 2:** Plasma lipid profiles in individuals that had undergone brain imaging (*n* = 24)

Plasma lipid	
Total cholesterol (mg/dl)	187.4 ± 27.9
Triglycerides (mg/dl)	116.0 ± 58.5
HDL cholesterol (mg/dl)	62 ± 30.4
LDL cholesterol (ng/dl)	107.7 ± 19.4

Data presented as mean ± standard deviation

### Plasma apoE levels differ between males and females

Both total plasma apoE (35.67 (16.84–137.52) μg/ml versus 32.58 (15.49–54.85) μg/ml, *p* = 0.0193) and apoE4 (5.03 (0.01–18.61) μg/ml versus 3.67 (0.15–11.37) μg/ml, *p* = 0.0267) levels were significantly higher in females (*n* = 91) versus males (*n* = 37), whereas plasma apoE3 levels did not significantly differ between the sexes. The overall apoE isoform distribution in total apoE did not differ between sexes with similar ratios of apoE3 and apoE4 to total apoE.

### Plasma apoE levels increase with age in females

The identified gender differences prompted us to examine potential apoE–age associations in females (*n* = 91) and males (*n* = 37) separately. Total apoE and apoE3 levels were weakly but significantly associated with age in females only (Spearman’s ρ = 0.2214, *p* = 0.0349 and Spearman’s ρ = 0.2264, *p* = 0.0309). Using an arbitrary cutoff point to divide older versus younger individuals into two groups (≤60 or ≥60 years of age), the older females displayed 11.5% higher total plasma apoE levels (*p* = 0.048) driven by an 11.7% increase in apoE3 levels (*p* = 0.045). Females aged 60 years or older exhibited 33% higher total apoE levels driven by an increase in apoE3 levels only, compared with their male counterparts (*p* = 0.001).

### Associations between plasma apoE levels, regional CMRgl and GMV

**Fig. 2:**
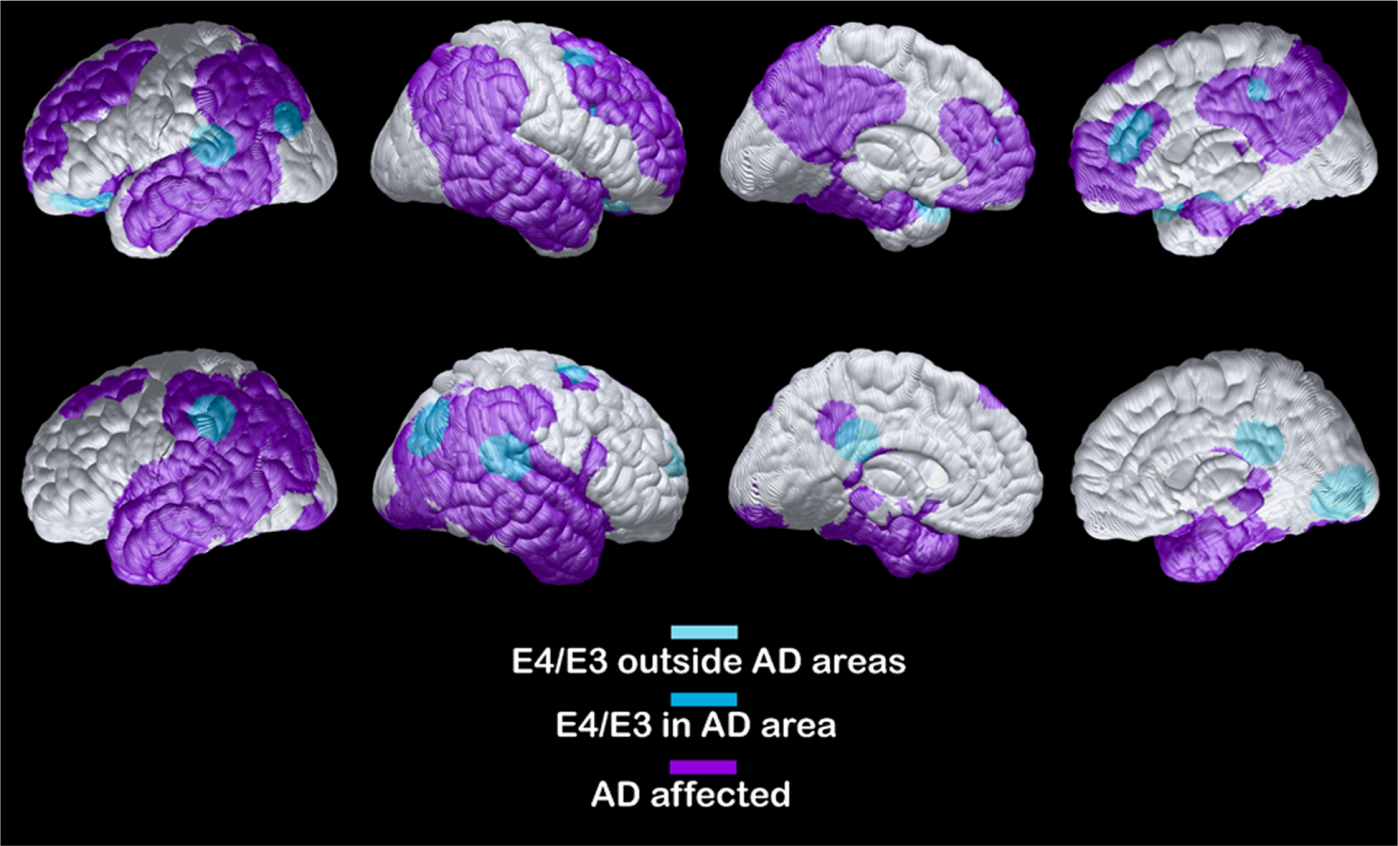
Negative associations between plasma apoE4/apoE3 ratio, CMRgl and GMV. Statistical brain maps depicting negative correlations (specific details in Table 3) between the plasma relative apoE4/apoE3 ratio and regional CMRgl (*top row*) and GMV (*bottom row*) in brain areas overlapping with (*blue*) or outside (*light blue*) areas commonly affected in AD (*purple*) [53] (Color figure online). *AD* Alzheimer’s disease

To assess whether peripheral apoE levels are associated with GMV and CMRgl, we consulted imaging data from 25 individuals who had undergone FDG-PET to determine CMRgl and MRI to determine regional GMV during the same visits as when blood samples were drawn. The characteristics of the individuals who underwent imaging are summarized in Table 1. Given our limited sample size and the potential number of comparisons we could make, we focused our examination on the association of the apoE4/apoE3 ratio with CMRgl and with GMV while also exploring such correlation with each apoE isoform. We first investigated whether the relative ratios between the apoE4 and apoE3 isoforms were negatively associated with regional CMRgl and GMV because numerous studies have attributed a gain of toxic function to the *APOE* ε4 gene product [22], and hence lower levels of this isoform may be beneficial. We found that higher apoE4/apoE3 ratios were indeed mainly associated negatively with measures on both the FDG-PET and MRI scans. Specifically, higher apoE4/apoE3 ratios were associated with glucose hypometabolism in the right hippocampus (Fig. 2 and Table 3). This *p* value survived multiple comparisons at the 0.05 level using SVC. A negative correlation was further observed between CMRgl and levels of the apoE4 isoform in the left inferior frontal orbital region (*p* = 0.000432), and a negative correlation with CMRgl and apoE3 isoform in the right frontal medial orbital (*p* = 0.00126) (Table 3). Similarly, higher apoE4/apoE3 ratios were negatively associated with GMV in the following common regions of GMV decline with AD: posterior cingulum, left hippocampus, left and right lateral temporal, left medial temporal and right precuneus (Fig. 2 and Table 3). Only the negative association of GMV and E4/E3 ratio in the posterior cingulum survived corrections at a 0.05 level with SVC.

**Table 3:** Correlations between regional glucose metabolism, gray matter volume and plasma apoE concentrations

apoE4/E3 ratio negative association	Brain region	Coordinates (*X*, *Y*, *Z*)	*p* value
CMRgl	Hippocampus_R	26, –12, –12	2.49 × 10^–4 a^
GMV	Cingulate_Post	4, –40, 21	5.60 × 10^–4 b^
	Hippocampus_L	–16, –34, 9	4.91 × 10^–3^
	Lateral Temporal_L	–48, –64, –3	2.81 × 10^–3^
	Lateral Temporal_R	60, –31, 13	1.83 × 10^–4^
	Medial Temporal_L	–16, –34, 9	4.91 × 10^–3^
	Precuneus_R	8, –43, 18	1.87 × 10^–3^

^a^ Survived correction at a 0.05 level

^b^ Survived correction at a 0.059 level

*CMRgl* cerebral metabolic rate of glucose, *GMV* gray matter volume

### Neuropsychological test results and correlations with apoE levels

We next assessed whether the identified associations could be reflected in scores on neuropsychological tests assessing verbal comprehension, working memory, word retrieval, attention and arithmetic capabilities mainly connected to the frontal and temporal lobes. Neuropsychological test battery results from the entire Arizona *APOE* Cohort (in total *n* = 71 *APOE* ε4 homozygotes and *n* = 194 heterozygotes as well as *n* = 356 non-*APOE* ε4 carriers used as controls) were published previously [37].

We used multivariable regression analysis to assess potential associations between plasma total apoE, apoE isoform levels, apoE4/apoE3 ratios and test scores for global cognition as well as scores from neuropsychological tests assessing performance in four cognitive domains: memory, language, executive and spatial functions. We found no correlation between global cognition scores and apoE levels in the two cohorts (data not shown) and our assessment of associations between scores from individual cognitive domain testing and apoE isoforms all turned out essentially negative when correcting for multiple testing (Bonferroni, four cognitive domains) (data not shown).

With significant differences in apoE levels between males and females, we explored sex-dependent associations between apoE levels and cognitive test scores. Splitting the large group into females (*n* = 91) versus males (*n* = 37) we found two associations between plasma apoE levels and cognition scores that remained significant after corrections, in females only. Total plasma apoE levels were positively associated with scores on the revised Wechsler Adult Intelligence Scale similarities subtest (WAIS-R similar) (Spearman’s ρ = 0.3691, *p* = 0.0003, corrected *p* = 0.0012). This correlation was driven by plasma apoE3 levels which were positively associated with scores on the WAIS-R similar test (Spearman’s ρ = 0.3081, *p* = 0.003, corrected *p* = 0.0012). Hence, higher apoE (apoE3) levels were linked to better performance on the verbal reasoning (similarities) subtest of the WAIS-R test in females only.

## Discussion

Identification of *APOE* ε4 phenotypical characteristics in humans should promote experimental hypothesis-driven research linking pathological cell and molecular processes to *APOE* ε4. We recently reported that *APOE* ε4 carriers have decreased levels of plasma apoE driven by a specific decrease in the apoE4 isoform levels [33]. We speculated that peripheral alterations in apoE concentrations predispose *APOE* ε4 carriers to processes leading to AD rather than cause the disease because in our previous study we found no difference in apoE levels between patients and controls. Interestingly, one study found that total plasma apoE levels were negatively linked to memory and verbal memory in older individuals when correcting for *APOE* ε4 carrier status [44], and results from the ADNI [45] proposed that lower total apoE levels were associated with smaller hippocampal size and that the association was driven by *APOE* ε4 status. Whether these associations can be traced to individual apoE isoform concentrations rather than total apoE levels remains to be investigated. We here quantified total plasma apoE and individual apoE isoform levels in nondemented individuals with an *APOE* ε4/ε3 genotype. We were interested to see whether the protein levels of the apoE4 risk isoform versus apoE3 in plasma could be associated with phenotypical characteristics reported in *APOE* ε4 carriers [11]. Our results indicated that the relative distribution between the risk-isoform apoE4 and the apoE3 isoform is of importance because a higher relative ratio of apoE4/apoE3 was linked to glucose hypometabolism and reduced GMV, for instance in the posterior and anterior cingulate areas. Hypometabolism in the medial temporal and parietal areas was proposed previously to predict cognitive decline in cognitively normal individuals [46], and in a large study including 600 cognitively normal individuals hypometabolism was associated with amyloid pathology as assessed using Pittsburgh compound B (PiB)-PET [47]. It was further found that *APOE* ε4 carriers exhibited marginally lower metabolism specifically in the posterior cingulate compared with noncarriers and the authors proposed that the *APOE*-associated observations were mediated through Aβ plaque load (PiB retention). Previous studies have shown that *APOE* ε4 carriers of similar age to those investigated here exhibit significant amyloid pathology even in the absence of cognitive symptoms [5]. A major weakness of the current study is that the studied individuals did not undergo CSF sampling and hence CSF AD biomarkers levels as surrogate markers of amyloid and tau pathology were not available.

Exactly how the relative ratio of apoE4 over apoE3 isoform levels in plasma can be related to the described structural and metabolic differences in the brain is not clear and linking peripheral apoE levels to processes in the brain is controversial because peripheral apoE does not cross the blood–brain barrier (BBB) [48]. Of note, we found that females exhibited higher apoE4 isoform levels than males and that the apoE3 levels were positively associated with age in females only. Results reported previously from the prospective study of the Danish general population also demonstrated an age-associated increase in levels of apoE (total levels) in both females and males; however, the increase in males appeared to plateau earlier than the increased levels found in females [34]. There could be several causes of the age-associated increase of apoE3 but not apoE4 that we observed in females, including altered levels of various sex hormones due to menopause; however, the lack of effect on the levels of apoE4 proposes differential effects of sex on different apoE isoforms. We speculate that an increased relative ratio of apoE4 over apoE3 may be ‘risky’ and that the levels of apoE3 are age-dependently upregulated as a protective feature in females who generally are at higher risk of AD [49]. Intriguingly, the specific role of apoE3 in *APOE* ε3/ε4 carriers is not well understood. The risk of developing AD is known to be higher in *APOE* ε3/ε4 carriers than in *APOE* ε2/ε4 carriers [3] and also the lipid and receptor binding capacities differ between the apoE isoforms [50]. If assuming a loss of function of the apoE4 isoform, a compensatory upregulation of the non-apoE4 isoform—apoE3 in the case of the herein studied individuals—may be beneficial. A full compensatory mechanism appears not to be present, however, as indicated by our previous findings showing lower plasma total apoE levels in *APOE* ε3/ε4 carriers versus *APOE* ε3/ε3 carriers [33]. Overall low total apoE levels were shown to increase the risk of AD and of other types of dementia in the Danish general population [34], but the question remains whether increased levels of all isoforms including apoE4 may be beneficial or not. Future studies further need to address whether the described age-associated and sex-associated effects also apply to apoE levels in CSF, in which we have previously shown that the ratio between the different apoE isoforms in total apoE differ from those in plasma [33].

In relation to the effect of sex, when exploring whether the variation in plasma apoE could be linked to cognitive performance in four major domains (memory, language, executive and spatial functions) we found only two weak correlations which were observed in females only. Hence as expected when studying a cohort that is cognitively intact (with little variation in cognitive performance), we did not find any strong correlations between plasma apoE levels and neuropsychological test results; however, our data do suggest the presence of a gender-specific effect on these correlations. Because of our limited sample size, future studies of larger cohorts are necessary to assess the specificity and significance of our observations linking plasma apoE levels to imaging findings and neuropsychological test outcomes.

The observed low levels of the apoE4 isoform with regard to total apoE levels are in line with our results reported previously [33]. However, the differences in apoE isoform levels between the sexes as well as an age-related increase in apoE3 in females only were not observed in our previous study [33]. Differences in cohort size and age of the included individuals may be factors that have influenced the outcome of the two studies. The lack of correlation between the apoE3 and apoE4 isoforms in plasma was surprising and needs further attention. It has been shown that the turnover rate differs between different apoE isoforms in plasma, with the apoE4 turnover rate almost 4-fold faster than that for apoE2 in *APOE* ε2/ε4 individuals [31]. As discussed before [33], it is not clear whether the apoE4 isoform levels result from fast degradation paralleled by a lack of compensatory upregulation of apoE4 synthesis or whether the synthesis of apoE4 per se is lower than that of other apoE isoforms due to differences at the expression and/or translation level in the periphery. Given our current results and previous results of a specific reduction in the apoE4 isoform in *APOE* heterozygous individuals [33], further studies are necessary to confirm that the reported differences are not due to alterations in the allele-specific expression of apoE.

## Conclusions

The current study provides the first evidence linking plasma levels of the individual apoE3 and apoE4 isoforms to structural and metabolic changes in the brains of cognitively intact *APOE* ε3/ε4 carriers. Specifically, the relative ratio between plasma levels of apoE4 and apoE3 appears to be of importance. Our study further provides new insights into sex-dependent differences in plasma apoE levels, with females exhibiting higher levels of the apoE4 isoform than males and increasing plasma apoE3 concentrations as a function of age. The exact relevance of these findings needs to be determined because female *APOE* ε4 carriers are at higher risk of AD than males [51, 52]. We conclude that peripheral levels of apoE and the isoform composition of total apoE levels are important features to consider with regard to risk and etiology of AD in carriers of the *APOE* ε4 risk allele.

## Additional file

Additional file 1: Table S1. Neuropsychological test data. (DOCX 13 kb)

## Abbreviations

AD: Alzheimer’s disease
ADNI: Alzheimer’s Disease Neuroimaging Initiative
apoE: Apolipoprotein E
BBB: Blood–brain barrier
CMRgl: Cerebral metabolic rate of glucose
DLB: Dementia with Lewy bodies
FDG-PET: Fluorodeoxyglucose positron emission tomography
GMV: Gray matter volume
HSPG: Heparin sulfate proteoglycan
MCI: Mild cognitive impairment
MRI: Magnetic resonance imaging
PiB-PET: Pittsburgh Compound-B positron emission tomography
SRM: Selected reaction monitoring
