# Supplementary material for: Peripheral apoE isoform levels in cognitively normal APOE ε3/ε4 individuals are associated with regional gray matter volume and cerebral glucose metabolism
Source: Alzheimers Res Ther. 2017 Jan 30;9:5. doi: 10.1186/s13195-016-0231-9 (PMC5282900; doi:10.1186/s13195-016-0231-9)
Supplement: Additional file 1: Table S1. — Neuropsychological test data. (DOCX 13 kb) [file 13195_2016_231_MOESM1_ESM.docx]

Table S1.

| Neuropsychological Test Scores | Imaged subjects (N=25) | Total  (N=128) |
| --- | --- | --- |
| ***Global Assessment***  Mini Mental State Examination-total score  Dementia Rating Scare  Instrumental Activities of Daily Living | 30(27-30) 0(0-0.5) 8(8-8) | 30(25-30) 0 (0-0.5) 8(8-9) |
| ***Memory*** Auditory Verbal Learning test-total learning score  Auditory Verbal Learning test-short term memory score  Auditory Verbal Learning test-long term memory score  Selective reminding test-free  Selective reminding test-cued  Rey-Osterrieth Complex Figure test – absolute recall  Benton Visual Retention Test – total number correct | 48(28-70) 10(4-15) 10(2-15) 98(66-111) 14(1-46) 20(7.5-34) 7(3-10) | 48(20-72) 10(1-15) 10(0-15) 96(35-112) 16(0-77) 20.5(0-34) 7(2-10) |
| ***Executive***  Wisconsin Card Sorting test – categories completed  Wisconsin Card Sorting test – total error  Wisconsin Card Sorting test – perseverative errors  Paced Auditory Serial Attention test task 3 – total correct  Paced Auditory Serial Attention test task 2 – total correct  Controlled Oral Word Association Test – total words  Wechsler Adult Intelligence Scale digit span subtest  Wechsler Adult Intelligence Scale mental arithmetic subtest  Wechsler Adult Intelligence Scale digit symbol substitution subtest | 6(0-6) 28(8-78) 16(4-45) 55(8-60) 46(23-60) 49(32-70) 11(6-19) 12(6-17) 13(7-18) | 6(0-6) 28(6-87) 13(4-55) 52.5(0-60) 43(0-60) 47(21-81) 11(5-19) 12(6-17) 13(7-19) |
| ***Language***  Boston Naming test (60 item) – total spontaneous correct  Token test – total correct  Wechsler Adult Intelligence Scale -R Vocabulary subtest  Wechsler Adult Intelligence Scale -R Similarities subtest | 58(44-60) 44(34-44) 13(10-18) 13(9-19) | 57(8-60) 44(34-44) 13(7-18) 13(9-19) |
| ***Spatial***  Judgement of Line orientation test – total correct  Facial Recognition test  Rey-Osterrieth Complex Figure test – copy score  WAIS-R Block design subtest | 25(20-30) 47(41-54) 36(21-36) 12(9-19) | 26(16-30) 47(30-54) 36(18-36) 12(4-19) |
